# Tuberculosis Knowledge, Attitudes, and Practice in Middle- and Low-Income Countries: A Systematic Review

**DOI:** 10.1155/2023/1014666

**Published:** 2023-06-24

**Authors:** Oana Marilena Craciun, Malen del Rosario Torres, Agustín Benito Llanes, María Romay-Barja

**Affiliations:** ^1^National Centre of Tropical Medicine, Institute of Health Carlos III, Madrid, Spain; ^2^Andrés Isola Hospital, Puerto Madryn, Chubut, Argentina; ^3^Centro de Investigación Biomédica en Red de Enfermedades Infecciosas (CIBERINFEC), Madrid, Spain

## Abstract

Tuberculosis (TB) is the leading cause of death from an infectious agent in the world. Most tuberculosis cases are concentrated in low- and middle-income countries. The aim of this study is to better understand tuberculosis-related knowledge about TB disease, prevention, treatment and sources of information, attitudes towards TB patients and their stigmatization and prevention, diagnosis and treatment practices in the general population of middle- and low-income countries, with a high tuberculosis burden, and provide evidence for policy development and decision-making. A systematic review of 30 studies was performed. Studies reporting on knowledge, attitudes, and practices surveys were selected for systematic review through database searching. Population knowledge about TB signs and symptoms, prevention practices, and treatment means was found inadequate. Stigmatization is frequent, and the reactions to possible diagnoses are negative. Access to health services is limited due to difficulties in transportation, distance, and economic cost. Deficiencies in knowledge and TB health-seeking practices were present regardless of the living area, gender, or country; however, it seems that there is a frequent association between less knowledge about TB and a lower socioeconomic and educational level. This study revealed gaps in knowledge, attitude, and practices in focused in middle- and low-income countries. Policymakers could take into account the evidence provided by the KAP surveys and adapt their strategies based on the identified gaps, promoting innovative approaches and empowering the communities as key stakeholders. It is necessary to develop education programs on symptoms, preventive practices, and treatment for TB, to reduce transmission and stigmatization. It becomes also necessary to provide communities with innovative healthcare solutions to reduce their barriers to access to diagnosis and treatment.

## 1. Introduction

Tuberculosis (TB) is a bacterial disease caused by mycobacterium tuberculosis that is spread through sputum when an infected person coughs, spits, or sneezes [1]. Every year 1.5 million people die from TB, making it the leading cause of death from an infectious agent worldwide. Most TB cases are concentrated in low- and middle-income countries [1]. Geographically, most people who developed TB in 2019 were in the WHO regions of South-East Asia (44%), Africa (25%), and the Western Pacific (18%) [2].

TB is a preventable and treatable disease mainly through promoting early screening and treatment for active TB, by addressing comorbidities and health risks as well as social determinants of the disease, and by promoting access to health care [3]. TB is a disease of poverty, and people affected by TB often face economic distress, vulnerability, marginalization, stigma, and discrimination [2]. Lack of awareness and insufficient knowledge about the symptoms of tuberculosis lead to a delay in seeking health care [4]. Therefore, disease control is improved, and treatment outcomes rely on improving awareness regarding TB [5].

To assess the awareness about TB, the best tool used is the knowledge, attitudes, and practice (KAP) survey. This is a representative study of a specific population to assess what is known, believed, and done in relation to TB [6].

KAP surveys can identify common knowledge gaps, cultural beliefs, or behavioral patterns that may facilitate or pose problems for TB control efforts. These surveys address key barriers to accessing TB care and completing treatment. The data collected provides government TB programs with the fundamental information needed to make strategic decisions [6].

Despite the fact that most of the cases are found in South-East Asia, most systematic reviews about tuberculosis' KAP surveys conducted in this region were addressed to healthcare workers [7, 8] while those addressed to the general population were focused on specific issues such as stigma [9], adherence to treatment [10], or centered in a limited geographical area [11]. The aim of this study was to better understand TB-related knowledge about disease, prevention, treatment, and sources of information; attitudes towards TB patients and their stigmatization and prevention, diagnosis and treatment practices, stigma-related attitudes; and prevention, diagnosis and treatment-related practices doing a systematic review of published KAP studies addressed to the general population of middle- and low-income countries, where 80% of cases and deaths are found [1], in order to provide evidence that could help policy strategies and decision-making.

## 2. Methods

### 2.1. Search Methods

A systematic review was conducted following the Preferred Reporting Items for Systematic Review and Meta-Analyses (PRISMA) guidelines and checklist [12] to identify evidence about knowledge, attitudes, and practices related to TB (see Table S1). Only peer-reviewed and published journal articles were reviewed. Literature search was performed in PubMed, Embase, and Scielo using Medical Subject Heading (MeSH) Search terms, and free language terms were used to maximize retrieval of potentially relevant studies. “Tuberculosis,” “Health Knowledge, Attitudes and Practices” terms were used. The search strategy is presented in Table S2.

### 2.2. Eligibility Criteria

To be eligible, articles have had to include research implemented in middle- or low-income countries according to the World Bank Classification [13]. Studies were also included if they were original research using quantitative knowledge, attitudes, and practices surveys or mixed methods. Studies could be published in Spanish, English, French, or Portuguese. The search was limited to articles published between January 1, 2010, and January 1, 2021. Articles were excluded if they did not use a KAP questionnaire, and the study participants were health professionals or medical students if the article was an opinion piece, conference abstract, or other systematic reviews.

### 2.3. Quality Assessment

The quality of the included studies was assessed with the NIH quality assessment tool for cohort, and cross-sectional studies [14] modified for cross-sectional studies. We assessed the representativeness of the study population, participation rate, correct recruitment, sample size justification, correct outcome definition and assessment, and confounding variables measurement. Each study included was assessed independently by two reviewers and was assigned a quality score ranging from 0 to 9. Quality was rated as 0 for poor (0–2 out of 9 points), + for fair (3–6 out of 9 points), or ++ for good (7–9 out of 9 points). While for the included studies with a mixed methods design, the mixed methods appraisal tool (MMAT) [15] was used to assess their quality, considering whether each study justified the rationale for the design, whether they integrated and interpreted quantitative and qualitative results, whether they explained any inconsistencies found, and whether they assessed the adequacy of the qualitative and quantitative components.

### 2.4. Data Collection and Analysis

Two reviewers conducted the search independently using the same eligibility criteria to evaluate the studies. Study data were extracted by each reviewer and verified by both of them. Conflicts were solved by a discussion with a third reviewer. After agreeing on the full-text eligible articles, each reviewer completed the information collection sheet including the name of the first author, year of publication, country and setting (urban/rural/both), sample size, survey location (home/community/hospital/health center), the aim of the study and outcomes classified by knowledge, attitudes, and practices reported in the survey by the participants. First, we have made a descriptive analysis of the frequencies of the studies settings (region, country, rural/urban, survey location), type of survey (quantitative or not), sample size, target population and subject (knowledge, attitudes, practices), and year of publication of each survey and summarized them in totals, percentages, and mean. We also summarized the responses collected in each study by subject (knowledge, attitudes, or practices) reporting their totals and percentages.

## 3. Results

**Figure 1:**
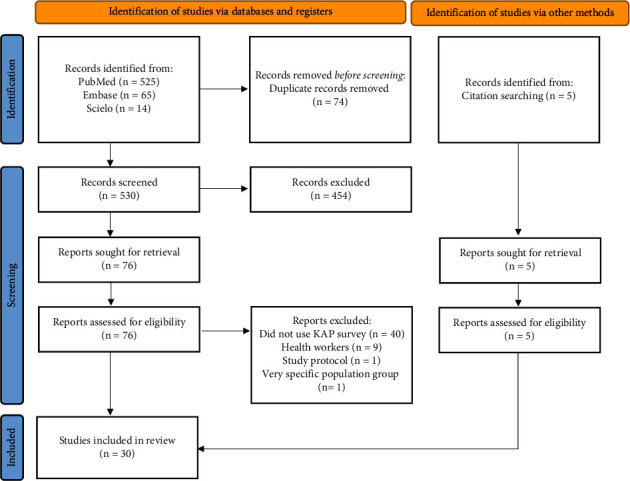
Flow diagram of the selection of studies for the systematic review.

Six hundred and four (*N* = 604) records were identified through a search in the aforementioned databases. Twenty-five were the articles included in the qualitative reading after full-text assessment for eligibility and, five records that met the inclusion criteria were added after being identified in the references of completed read articles. Finally, a total of thirty (*N* = 30) articles were included (Figure 1).

Most of the studies reviewed were conducted in Africa (56.7%) (Table 1). Of the 30 studies, all of them were cross-sectional studies, 24 were quantitative studies, and 6 were mixed, all of them used the knowledge, attitudes, and practices (KAP) questionnaire as a survey tool. The studies were mainly addressed to the general population (63.4%). The mean sample size was 1557, and the median sample size was 699.

Table 2 shows a detailed summary of the study settings, aims, and outcomes of reviewed studies.

### 3.1. Knowledge about TB

All of the 30 studies included provided information on the responses of their participants regarding knowledge.

#### 3.1.1. Causes

The most frequent correct cause of TB mentioned was “germs” in 23% of the included studies [19, 21–24, 31, 42]. Other causes frequently mentioned were misconceptions like “drinking raw milk” or “eating contaminated food” “witchcraft,” “exposure to dust,” “exposure to cold” or “smoking” and “hereditary illness” [17, 19, 22, 25, 28, 39, 40, 42, 45] (Table 3).

#### 3.1.2. Signs and Symptoms

Less than half of the studies collected correct answers about TB signs and symptoms (Table 3). The most commonly correct symptoms mentioned were “cough for 2 weeks or more,” “cough,” “weight loss,” “hemoptysis,” “chest pain,” “fever,” “fatigue” and “shortness of breath.” The most incorrectly mentioned symptoms were “nausea” and “headache,” and others such as “hair color changes,” “skin infections” or “joint pain” were also included [19, 22, 25–28, 31–33, 35–37, 39, 40, 43, 45].

#### 3.1.3. Mode of Transmission

Population knowledge about TB modes of transmission was also found insufficient in 20% of the studies [18, 21, 30, 31, 38, 44]. The correct modes of transmission most mentioned were “via respiratory routes” and “living with a TB patient” [17, 19–24, 26, 27, 30–33, 36, 39–45]. The incorrect modes of transmission were “sharing meals or plates,” “handshakes” or “skin contact,” “needles,” “having sex with a woman who miscarried,” “food poisoning,” “sleeping with a widow,” and “touching public items” (Table 3) [20, 22, 26, 27, 32, 33, 36–38, 43, 45].

#### 3.1.4. Prevention

The results regarding prevention methods were very diverse, and the most common were “covering the mouth and nose,” “BCG vaccine,” and “treatment” [18, 22, 25, 26, 32, 36, 37, 39, 42, 45]. The incorrect prevention methods most mentioned were “separating dishes,” “good hygiene and washing hands,” and “closing windows” [19, 22, 25, 26, 32, 36, 37, 39, 45]. Also in one study, the authors of [22] mentioned “no smoking” and “avoid sex” or “no spitting” [39].

#### 3.1.5. Knowledge about TB Cure

When asked about if TB can be cured, 37% of the studies showed that the population believed that TB can be cured [16, 20, 24, 25, 27–29, 32, 34, 35] (Table 3). Only four studies mentioned that the population believed that TB cannot be cured, and two of them [18, 22] described that a high percentage of their study population believed that TB has no cure and is a killer disease even after treatment.

#### 3.1.6. TB Treatment, Treatment Cost, and Best Place

The reviewed literature showed that the majority (53%) of the studies reported that their population knew that TB is a treatable disease although in many cases, they have insufficient knowledge about treatment means [16, 17, 19, 22, 25, 27, 33, 34, 36, 37, 39, 40, 42–45].

Regarding knowledge about TB treatment, the most frequently mentioned in the studies was “modern drugs” or “modern treatments” by 50% of the studies [16, 17, 19, 22, 25–27, 33, 34, 36, 37, 39, 42, 44, 45]. However, misconceptions and traditional treatments were also frequently mentioned. The most common incorrect forms of treatment mentioned were “traditional medicine,” “herbal remedies,” and “roots, blood, skin cuts,” and other incorrect treatments mentioned were “resting,” “religious treatment,” and “burning swelling sites with heated metals” [22]. Only two studies [25, 27] mention that a part of their population did not know any treatments for TB.

In relation to the cost of treatment, 40% of the articles mentioned that the population knew that the treatment is “free of any charges” [17, 19, 21, 25, 32, 34–37, 39, 40, 43]. However, some mentioned, “not knowing the cost,” and even that the treatment was “not free” or “expensive” [17, 21, 25, 34, 36, 37].

Regarding the knowledge of the duration of treatment some studies mention different lengths of time “6 months” [36, 44], “more than 6 months” [36], and “less than 6 months” [36, 44].

When asked about the place where to obtain treatment, 17% of the studies conducted in Africa reported “health centers” and “traditional healers” in a study from Asia [36] (Table 3).

#### 3.1.7. Sources of Information about TB

The most common sources from which the population successfully obtained information on TB described in most articles were “Television (TV),” “Health Extension Workers (HEWs),” “Radio,” and “Family and/or Friends” [16, 18, 21, 22, 24–27, 30–33, 35–37, 42, 43, 45]. The sources less mentioned or mentioned to be less effective as TB information sources were “Internet” [33, 35], “magazines” or other written sources [21, 27, 33, 37] and “religious leaders” [21, 37]. The preference in sources of information on TB according to the area of study was HEWs in rural areas and TV in urban areas.

### 3.2. Attitudes regarding TB

#### 3.2.1. Stigma

Stigmatization towards TB patients was present in 20 (67%) of the included studies, showing high levels of stigmatization in 37% of them “more than 50% of the population feels rejection towards TB patients, would not support them, and think that TB is an illness that one should be ashamed of” [17, 22, 23, 25, 35, 36, 39, 41, 42, 44, 45]. It is important to highlight that many of these studies were conducted only in rural areas [17, 22, 23, 25, 44] while the studies that presented lower levels of stigmatization were conducted mostly in urban areas [19, 21, 26, 31] or in both [32, 33, 36, 38].

#### 3.2.2. Reaction to TB Diagnosis

When asked about the participants' reactions to TB diagnosis in the selected studies, the answers were “fear,” “shame,” and “sadness and hopelessness” in 8 (27%) of the included studies [22, 24–26, 33, 36, 37, 39, 42, 45].

In addition, only 3 studies showed that some of the participants would be willing to reveal having TB to their families or friends [30, 34, 42].

### 3.3. Practices and Health-Seeking Behavior

#### 3.3.1. Health-Seeking Practices

Of the included studies, 20% reported that the majority of the studied population said that they used to go to health centers or the Community Health Worker (CHW) to seek health care at least once a year [19, 25–27, 34, 42]. Therefore, when asked what they would do if they had symptoms of TB, the most common answers were “go to a health care facility,” “go to the Community Health Worker (CHW),” or “go to the hospital” [16, 19, 22, 24–26, 30, 32, 33, 36, 37, 42, 44, 45] although 9% of the studies showed that most participants only would go to the hospital when traditional or self-treatment failed and the symptoms were persistent or incapacitating [27, 37, 45] (Table 4).

The incorrect health-seeking practices mentioned in the reviewed studies were “self-treatment” (27%), “traditional healer” (20%), and “going to the pharmacist” (13%) [16, 23, 25–28, 30, 33, 36, 37, 42, 45]. Only one study [29] mentioned that poor health behavior might generate drug resistance to TB.

#### 3.3.2. Delay

Out of the 30 selected studies, only 2 (7%) analyzed that the average delay of treatment seeking among the population was 7 days after symptoms onset, one in a rural area of Asia, Myanmar [44] and one in rural Africa, Ethiopia [25].

#### 3.3.3. Access Barriers

When asked about the difficulties found in attending healthcare facilities, the participants of the reviewed studies mentioned “economic cost,” “distance,” “transportation,” “not knowing where TB treatment could be accessed,” “difficulty taking time off from work,” and “fear of stigma” [19, 25, 28, 30, 33, 44] (Table 4).

#### 3.3.4. Prevention Practices

Five studies (17%) mentioned some prevention practices that the respondents were actively using, such as “avoid sharing food utensils” [22], “covering the mouth and nose when coughing” [21, 31], “maintaining a correct ventilation of the house” [21, 31], or “limiting contact with TB patients” [40]. Only one study [19] mentioned that a small percentage of the participants answered that TB could not be prevented (Table 4).

### 3.4. Critical Appraisal

All the included studies had fair or good quality after the quality assessment. All the studies had a clearly stated research question and a specified study population. The main limitations of the included studies were related to the reporting of the sampling strategy, the participation rate, and the qualitative results in some of the studies with mixed methods. The complete quality assessment tables are available in Table S3.

## 4. Discussion

Tuberculosis is a major cause of ill health, one of the top 10 causes of death worldwide and the leading cause of death from a single infectious agent [1]. TB treatment has prevented more than 60 million deaths, but many millions have also been left without diagnosis and care [2].

India, Indonesia, the Philippines, and South Africa are the four countries that account for 44% of global TB cases [2]. However, only 7 of the included studies (23%) were conducted in South-East Asia, highlighting a gap in KAP on TB research in the region.

Studies results showed how the awareness about TB in the general population of middle- and low-income countries is still insufficient. These deficiencies implied inadequate attitudes and practices, causing delay in seeking treatment and increasing transmission [4, 46, 47].

Our review shows that inadequate knowledge and TB health-seeking practices were present regardless the gender, living area (urban or rural), or country. In addition, it seems that there is a frequent association between less knowledge about TB and a lower socioeconomic and educational level [47, 48]. Low income and low education populations are more likely to have incorrect perceptions of TB, such as more incorrect responses to the causes, symptoms, and transmission and prevention. Low awareness about TB associated with being unemployed or not have completed high school education was also found in other two systematic reviews [47, 48].

The respondents knew that the best place to go to treat TB is the health services, where they could be treated with “modern drugs”. However, this is not reflected in their practices as they preferred to go to the traditional healers or even used self-treatment [17, 27, 34, 39]. Barriers in accessing health services are among the most mentioned reasons why individuals did not frequent them when they present symptoms, as shown in a study conducted in Kenya where distance to the health centers was a factor in underdiagnoses and poorer treatment outcomes [49]. The training of community health workers and the promotion of diagnosis in peripheral and rural areas could be recommended, as a way of bringing health care closer to the most remote areas.

The presence of TB disease is often believed to be an event of which one should be ashamed of. Stigmatization of TB patients is found more frequent in rural areas than in urban areas. In Ethiopia, TB patients may deliberately hide their health status or try to live with TB without treatment to avoid stigma, being the source of infection to others [50]. In Nepal, TB is also a source of stigmatization and isolation, causing delays in seeking healthcare and treatment [51]. Delay in seeking treatment is among the most common practice that could lead to the spread of the disease in the community. The most common causes for delay in seeking TB care found were difficulties in transportation and lack of knowledge about where to search for treatment [25].

The preferred sources of information about TB in this review were TV in urban and HEWs in rural areas. Studies have shown that mass media is an adequate source of information about TB [52, 53]. Furthermore, more exposure to mass media when used to give information about TB translates into better knowledge and attitude, being TV the most popular mass-media source [53, 54]. A study carried out in India showed that the rural population or those with a lower socioeconomic level have less access to the mass media and subsequently less access to information about TB [55], recommending that HEWs would be the most appropriate source of information for the rural population.

The quality of evidence from some of the included studies was overall acceptable; yet, the main limitations of the included studies were that some did not report the rate of participation, and some studies did not explain the estimation of the sample size. Also, due to the inclusion criteria, there is little presence of studies conducted in the SEA region. Notwithstanding these limitations, the need to improve TB awareness and health-seeking behavior showed evident.

## 5. Conclusion

This study revealed gaps in TB knowledge, attitude, and practices in the general population in middle- and low-income countries. The evidence shown in this study should be considered to improve education programs on symptoms, preventive practices, and TB treatment in order to reduce stigma and the barriers associated with diagnosis and treatment access, using mass media in urban areas and HEWs in rural areas as sources of information, focusing on the most vulnerable population. Furthermore, successful control of TB relies on coherent, multifaceted community-based health programs. Policymakers should take into account the relevant information provided by KAP surveys and adapt their strategies to evidence, promoting innovative approaches such as new mechanisms for drug distribution and diagnosis and empowering the communities as key stakeholders. Implementing community-directed programs would provide opportunities for government health services and other local social actors to work closely with the population directly affected, reaching as many people as possible, and taking into account socio-cultural and economic conditions.

## Figures and Tables

**Table 1:** Descriptive analysis of the 30 selected studies for qualitative analysis.

	*n*	(%)
*Region*		
Africa	17	56.7
America	2	6.7
Asia	11	36.6

*Study country*		
Angola	1	3.3
Botswana	1	3.3
Cameroon	1	3.3
Colombia	2	6.7
Ethiopia	6	20.0
India	4	13.3
Iran	1	3.3
Madagascar	2	6.7
Mongolia	1	3.3
Myanmar	1	3.3
Nigeria	4	13.3
Pakistan	3	10.0
Sudan	1	3.3
Tajikistan	1	3.3
Tanzania	1	3.3

*Study type*		
Quantitative	24	80
Mixed	6	20

*Study population*		
General population	19	63.4
Women	3	10.0
TB patients	1	3.3
Other	7	23.3

*Sample size*		
Mean	1556.6	
Median	699	
Mode	422	

*Study design*		
Transversal	30	100

*Study scope*		
District	23	76.7
National	7	23.3

*Study environment*		
Rural	10	33.3
Urban	7	23.3
Both	13	43.4

*Survey place*		
Home	19	63.4
Hospital	1	3.3
Healthcare center	2	6.7
Community	4	13.3
Other	4	13.3

*Language*		
English	29	96.7
Spanish	1	3.3

*Subject*		
Knowledge	2	6.7
Knowledge and attitudes	2	6.7
Knowledge and practices	2	6.7
Full KAP	24	79.9

Mean year of publication	2014	

**Table 2:** Summary of the study settings, aims, and outcomes of the included studies for this systematic review (*n* = 30).

Study ID	Country and setting	Sample size	Survey location	Aim of the study	Outcomes		
					Knowledge	Attitudes	Practice
[16]	Sudan, urban-rural	1275	HC^†^	Contribute to knowledge about barriers to the success of the TB^§^ control program and inform program managers and decision-makers	Symptoms, transmission, prevention, level of knowledge, sources of information about TB and cure		Healthcare seeking and treatment
[17]	Nigeria, rural	1186	Home, community	Determine the level of KAP^‡^ of people towards TB in a rural Nigerian community	Cause, symptoms, transmission, prevention, level of knowledge, and treatment	Stigma, beliefs, and screening test	Treatment
[18]	India, rural	128	Hospital	Investigate TB KAP among HIV-infected patients in a high HIV prevalence region of India	Transmission, sources of information about TB and cure		
[19]	Nigeria, urban	916	Home	Reduce the burden of TB disease in 18 communities through educational intervention, TB case detection and integration into the State National Tuberculosis and Leprosy Control Program (NTBLCP)	Cause, symptoms, transmission, prevention, and treatment	Stigma and beliefs	Healthcare seeking, treatment, difficulties accessing healthcare and prevention
[20]	Iran, urban	593	Other (street)	Define the KAP of homeless people in Tehran regarding TB and HIV	Transmission, level of knowledge, and cure		
[21]	Nigeria, urban	504	Home	Examine the current level of knowledge and attitude with the goal of guiding future interventions	Cause, symptoms, transmission, prevention, level of knowledge, sources of information about TB and treatment	Stigma, reaction to diagnosis, and beliefs	Prevention
[22]	Ethiopia, rural	422	Home	Assess communities' KAP regarding TB in South Western Ethiopia	Cause, symptoms, transmission, prevention, level of knowledge and sources of information about TB	Stigma and reaction to diagnosis	Treatment
[23]	Colombia, rural	300	Community	Describe the KAP on tuberculosis and its association with some sociodemographic aspects of inhabitants of rural and indigenous areas of Córdoba (Colombia) and evaluate the validity and reliability of the KAP scale	Cause, symptoms, transmission, prevention and sources of information about TB	Stigma, beliefs, and screening test	Healthcare-seeking and nearby cases
[24]	Ethiopia, urban-rural	3503	Home	Better understand TB-related knowledge, attitudes, and practices (KAPs) and generate evidence for policy and decision-making	Cause, symptoms, transmission, level of knowledge, sources of information about TB and cure	Reaction to diagnosis	Healthcare seeking
[25]	Ethiopia, rural	422	Hospital, HC	Evaluate of the knowledge, attitudes and health-seeking behaviors and associated factors of pulmonary tuberculosis patients	Cause, symptoms, prevention, level of knowledge, sources of information about TB and cure	Stigma, secret, and reaction to diagnosis	Healthcare seeking, delay, difficulties accessing healthcare
[26]	Colombia, urban	878	Home	Describe the KAP about TB in the HCs of patients diagnosed with this disease	Symptoms, transmission, prevention, sources of information about TB, cure and treatment	Stigma and reaction to diagnosis	Healthcare seeking
[27]	Tajikistan, rural	509	Home	Elucidating the key factors influencing access to TB diagnosis and treatment among migrants in their labor destination country and home country	Cause, symptoms, level of knowledge, sources of information about TB and cure	Stigma	Healthcare seeking and treatment
[28]	Tanzania, rural	105	Community	Evaluation of the KAP among the masai on TB and gain insight into the role of traditional healers	Cause, symptoms, and cure	Beliefs	Healthcare seeking, treatment, and difficulties accessing healthcare
[29]	Nigeria, urban-rural	3021	Home	Measure knowledge of TB among the general population	Level of knowledge and cure		Healthcare seeking and treatment
[30]	India, rural	1985	Home	Assess the level of awareness, attitude, and treatment-seeking behavior regarding tuberculosis in a rural area of Tamil Nadu	Transmission and sources of information about TB	Secret	Healthcare seeking and difficulties accessing healthcare
[31]	Ethiopia, urban	420	HC^†^	Assess knowledge, attitude, and preventive practice towards tuberculosis	Cause, symptoms, transmission, prevention, level of knowledge, and sources of information about TB	Stigma and beliefs	Prevention
[32]	Pakistan, rural	488	Home	Understand and assess the knowledge, awareness, perceptions, and health-seeking behavior of general and TB-affected population	Symptoms, transmission, prevention, sources of information about TB, cure, and treatment	Stigma	Healthcare seeking
[33]	Cameroon, urban-rural	3663	Home	Assess TB-related KAP in Cameroon and identify barriers to seeking care	Symptoms, transmission, and sources of information about TB	Stigma and reaction to diagnosis	1 2 4 healthcare seeking, treatment, and difficulties accessing healthcare
[34]	South Angola, urban-rural	805	Home	Measure health-seeking behavior in Southern Angola and to inform the design of context-specific interventions to improve case detection	Symptoms	Secret	Healthcare seeking and treatment
[35]	Mongolia, urban-rural	10581	Home	Estimate the level of KAP concerning TB among the public and to examine how those are affected by demographic, socioeconomic, and policy factors in inner Mongolia	Symptoms, transmission, level of knowledge, sources of information about TB and cure	Stigma	
[36]	Pakistan, urban-rural	1080	Home	Explore KAP regarding TB in the general population of two districts of Punjab province, and the effect of socioeconomic determinants	Symptoms, transmission, prevention, and sources of information about TB	Stigma and reaction to diagnosis	Healthcare seeking and treatment
[37]	Pakistan, urban-rural	1080	Home	Explore inequities in KAP regarding TB among the population	Symptoms, transmission, prevention, level of knowledge, sources of information about TB and treatment	Stigma, reaction to diagnosis, and beliefs	Healthcare seeking and treatment
[38]	Botswana, urban-rural	2032	Home	Assess KAP of communities on TB and identify sources of their information on this disease and HIV	Transmission	Stigma and beliefs	
[39]	Madagascar, urban	266	Home	Assess the KAP of the population for the management of the disease and propose recommendations to the authorities based on the study	Symptoms, transmission, prevention, level of knowledge and cure	Stigma and beliefs	Healthcare seeking and treatment
[40]	Madagascar, urban	68	Community	Evaluate KAP to the distribution of TB and to provide information for the improvement of the National Tuberculosis Program	Cause, symptoms, transmission, sources of information about TB and treatment	Beliefs	Treatment and prevention
[41]	India, urban-rural	4562	Home	Describe the prevalence of stigmatizing attitudes towards TB patients among general population and their association with knowledge regarding TB	Transmission and level of knowledge	Stigma	
[42]	Ethiopia, urban-rural	585	Home	Assess the pastoralist community KAP towards TB in comparison to the neighboring sedentary communities	Cause, transmission, prevention, level of knowledge, and sources of information about TB	Stigma, secret, reaction to diagnosis and beliefs	Healthcare seeking and treatment
[43]	India, urban-rural	4562	Home	Assess knowledge of TB and its association with respondent's sociodemographic characteristics	Symptoms, transmission, sources of information about TB and treatment	Screening test	Treatment
[44]	Myanmar, rural	349	Other (workplace)	Evaluate workers' KAP about TB	Transmission	Stigma	Healthcare seeking, treatment, delay, and difficulties accessing healthcare
[45]	Ethiopia, urban-rural	410	Home	Assess community's KAP towards TB	Cause, symptoms, transmission, prevention, level of knowledge, and sources of information about TB	Stigma, reaction to diagnosis and beliefs	Healthcare seeking and treatment

^†^HC: health center. ^‡^KAP: knowledge, attitudes, and practice. ^§^TB: tuberculosis.

**Table 3:** Summary of most common responses about TB knowledge and the studies that mention them.

Item	Adequate	*n* (%)	Inadequate	*N* (%)	Studies that mention this item
Causes	“Germs”	7 (23%)	“Drinking raw milk”	4 (13%)	[17, 19, 21–25, 28, 31, 39, 40, 42, 45]
			“Eating contaminated food”	3 (9%)	
			“Witchcraft”	3 (9%)	
			“Exposure to dust”	2 (7%)	
			“Exposure to cold”	3 (9%)	
			“Smoking”	2 (7%)	
			“Hereditary illness”	3 (9%)	

Signs and symptoms	“Cough for 2 weeks or more”	11 (37%)	“Nausea”	3 (9%)	[19, 22, 25–28, 31–33, 35–37, 39, 40, 43, 45]
	“Cough”	10 (33%)			
	“Weight loss”	10 (33%)	“Headache”		
	“Hemoptysis”	9 (30%)			
	“Chest pain”	5 (17%)	“Hair color changes”	2 (7%)	
	“Fever”	3 (9%)	“Skin infections”		
	“Fatigue”	3 (9%)	“Joint pain”		
	“Shortness of breath”	3 (9%)			

Mode of transmission	“Via respiratory routes”	17 (57%)	“Sharing meals or plates”	10 (33%)	[17, 19–24, 26, 27, 30–33, 37–45]
			“Needles”	1 (3%)	
			“Having sex with a woman who miscarried”	1 (3%)	
			“Food poisoning”	1 (3%)	
	“Living with a TB patient”	2 (7%)	“Sleeping with a widow”	1 (3%)	
			“Handshakes” or “skin contact”	6 (20%)	
			“Touching public items”	3 (9%)	

Prevention	“Covering the mouth and nose”	8 (27%)	“Separating dishes	5(17%)	[18, 19, 22, 23, 25, 26, 32, 36, 37, 39, 42, 45]
			“Good hygiene” or “washing hands”	4 (13%)	
	“BCG vaccine”	4 (13%)			
			“Closing windows”	4 (13%)	
	“Treatment”	3 (9%)	“No smoking”	1 (3%)	
			“Avoid sex”	1 (3%)	
			“No spitting”	1 (3%)	
			“Avoid touching”	1 (3%)	

Knowledge about cure	“TB can be cured”	11 (37%)	“TB is not a curable disease”	4 (13%)	[16, 18, 20, 24–29, 32, 34, 35, 39]

TB treatment	“Modern drugs”	15 (50%)	“Traditional medicine”	6 (20%)	[16, 17, 19, 21, 22, 25, 27, 28, 32–34, 36, 37, 39, 40, 42–45]
			“Herbal remedies”	5 (17%)	
			“Roots, blood, skin cuts”	1 (3%)	
	“Treatment is free of any charges”	12 (40%)	“Resting”	5 (17%)	
			“Religious treatment”	4 (13%)	
			“Burning swelling sites with heated metals”	1 (3%)	
	“Duration of treatment: 6 months”	2 (7%)	“Not knowing the cost of the treatment”	2 (7%)	
			“Expensive treatment”	5 (17%)	
			“Duration more/less than 6 months”	3 (9%)	

TB: tuberculosis. BCG: Bacille Calevette-Guerin.

**Table 4:** Practices related to TB prevention and diagnosis seeking.

Item	Adequate	*n* (%)	Inadequate	*n* (%)	Studies that mention this item
Health-seeking practices	“Go to a health care facility”	11 (37%)	“Self-treatment at home”	8 (27%)	[19, 22–27, 30, 32–34, 36, 37, 42, 45]
	“Go to CHW”	1 (3%)	“Traditional healers”	6 (20%)	
	“Go to the hospital”	5 (17%)	“Going to the pharmacist”	4 (13%)	

Prevention practices	“Covering mouth and nose when coughing”	2 (7%)	“Avoid sharing food utensils”	1 (3%)	[19, 21, 22, 31, 40]
	“Correct ventilation of the house”	2 (7%)	“TB cannot be prevented”	1 (3%)	
	“Limiting contact with TB patients”	1 (3%)			

Difficulties accessing healthcare	“Economic cost”			4 (13%)	[19, 25, 28, 30, 33, 44]
	“Transportation difficulties”			2 (7%)	
	“Not knowing where TB treatment could be accessed”			2 (7%)	
	“Distance”			3 (9%)	
	“Unwelcoming care staff”			3 (9%)	
	“Difficulty taking time from work”			2 (7%)	
	“Fear of stigma”			1 (3%)	

TB: tuberculosis. CHW: community health worker.

## Data Availability

No data were used in this study.

## Supplementary Materials

Supplementary Materials Table S1. Reporting Items for Systematic Review and Meta-Analyses (PRISMA). Table S2. Search strategy. Table S3. Quality assessment of included studies using the Mixed Methods Appraisal Tools (MMAT).
