# Supplementary material for: Tuberculosis Knowledge, Attitudes, and Practice in Middle- and Low-Income Countries: A Systematic Review
Source: J Trop Med. 2023 Jun 24;2023:1014666. doi: 10.1155/2023/1014666 (PMC10314818; doi:10.1155/2023/1014666)
Supplement: Supplementary Materials — Table S1. Reporting Items for Systematic Review and Meta-Analyses (PRISMA). Table S2. Search strategy. Table S3. Quality assessment of included studies using the Mixed Methods Appraisal Tools (MMAT). [file 1014666.f1.zip › 1014666.f1/Table_S2.docx]

| **Search number** | **Query** | **Search Details** | **Results** |
| --- | --- | --- | --- |
| 1 | "Tuberculosis"[Mesh] | "Tuberculosis"[MeSH Terms] | 198,388 |
| 2 | "Health Knowledge, Attitudes, Practice"[Mesh] | "health knowledge, attitudes, practice"[MeSH Terms] | 120,425 |
| 3 | 1 AND 2 | "Tuberculosis"[MeSH Terms] AND "health knowledge, attitudes, practice"[MeSH Terms] | 824 |
| 4 | (("health workers") AND ("health personnel")) AND ("healthcare workers") | "health workers"[All Fields] AND "health personnel"[All Fields] AND "healthcare workers"[All Fields] | 305 |
| 5 | 3 NOT 4 | ("Tuberculosis"[MeSH Terms] AND "health knowledge, attitudes, practice"[MeSH Terms]) NOT ("health workers"[All Fields] AND "health personnel"[All Fields] AND "healthcare workers"[All Fields]) | 819 |
| 6 | 3 NOT 4 limited to 1/1/2010 to 1/1/2021 | (("Tuberculosis"[MeSH Terms] AND "health knowledge, attitudes, practice"[MeSH Terms]) NOT ("health workers"[All Fields] AND "health personnel"[All Fields] AND "healthcare workers"[All Fields])) AND (2010/1/1:2021/1/1[pdat]) | 525 |

**Table S2.** Search strategy for PubMed
