# Supplementary material for: Tuberculosis Knowledge, Attitudes, and Practice in Middle- and Low-Income Countries: A Systematic Review
Source: J Trop Med. 2023 Jun 24;2023:1014666. doi: 10.1155/2023/1014666 (PMC10314818; doi:10.1155/2023/1014666)
Supplement: Supplementary Materials — Table S1. Reporting Items for Systematic Review and Meta-Analyses (PRISMA). Table S2. Search strategy. Table S3. Quality assessment of included studies using the Mixed Methods Appraisal Tools (MMAT). [file 1014666.f1.zip › 1014666.f1/Table_S3 (1).docx]

**Table S3.** Quality assessment of included studies using the Mixed Methods Appraisal Tools (MMAT).

|  | **Criteria** | | | | | | |
| --- | --- | --- | --- | --- | --- | --- | --- |
| **Study** | S1. Are there clear research questions? | S2. Do the collected data allow to address the research questions? | 1. Is there an adequate rationale for using a mixed methods design to address the research question? | 2. Are the different components of the study effectively integrated to answer the research question? | 3.. Are the outputs of the integration of qualitative and quantitative components adequately interpreted? | 4. Are divergences and inconsistencies between quantitative and qualitative results adequately addressed? | 5. Do the different components of the study adhere to the quality criteria of each tradition of the methods involved? |
| Anochie et al., 2013 | Yes | Yes | Yes | CD | Yes | CD | Yes |
| Gilpin et al., 2011 | Yes | Yes | Yes | CD | Yes | Yes | Yes |
| Hassan et al., 2017 | Yes | Yes | CD | Yes | No | No | No |
| Khan et al., 2020 | Yes | Yes | Yes | Yes | Yes | Yes | Yes |
| Rakotosamimanana et al., 2014 | Yes | Yes | Yes | Yes | Yes | CD | Yes |
| Thu et al., 2012 | Yes | Yes | Yes | Yes | Yes | CD | Yes |
| CD: Cannot determine | | | | | | | |
